# Low Dose Naltrexone and Lung Cancer: A Case Report and Discussion

**DOI:** 10.7759/cureus.2924

**Published:** 2018-07-05

**Authors:** Jeffrey A Miskoff, Moiuz Chaudhri

**Affiliations:** 1 Medicine, Jersey Shore University Medical Center, Neptune City, USA

**Keywords:** low dose naltrexone, chron's disease, non-small cell lung cancer, neurodegenerative conditions, autoimmune disorders, human immunodeficiency virus, endorphins, proclaim trial, metkephalin

## Abstract

Low dose naltrexone (LDN) has been promising as a complementary medication for patients with a broad range of medical disorders. Although not a proven cure, evidence from clinical trials supports LDN as being a valuable adjunct for disorders in which the immune system plays a centralized role. Additionally, clinical trials have proposed a unique mechanism(s) allowing LDN to affect tumors including non-small cell lung cancer (NSCLC) at the cellular level by augmenting the immune system. We present a case of a 50-year-old male with a prolonged survival and a past medical history of prostate and lung cancer.

## Introduction

Naltrexone has been an option for managing patients with alcohol or opioid dependence as it has been approved by the Food and Drug Administration (FDA) in 1984. Low dose naltrexone (LDN) has been gaining credibility in its ability to halt the progression of several diseases without significant side effects when administered in low dosage. Furthermore, LDN has produced positive results in clinical trials taking place throughout the USA and other countries. A double-blind, placebo-controlled multiple sclerosis (MS) trial at the University of California, San Francisco (UCSF) concluded that the patients on LDN showed a significant improvement (3.3 points) on the following Mental Component Summary Score of the 36-item Short Form Health Survey (SF-36) [1]. Also, a HIV trial in Mali concluded that the patients who were diagnosed with HIV when treated with antiretroviral drugs (ARV) and LDN showed a significant improvement in their cluster of differentiation (CD4) count after six months of therapy compared to the individuals who were given ARV and placebo regimen [2]. Finally, a randomized, double-blind placebo-controlled study at the Penn State College of Medicine (PSU) focusing on Crohn’s disease (CD) determined that 88% of the patients receiving LDN versus the control group had a 70-point decline in Crohn’s Disease Activity Index Score (CDAI) [3-4]. 

Low dose naltrexone possesses a unique mechanism allowing it to briefly block the endogenous opioid production creating a transient opioid deficit. Thus, it stimulates the hypothalamus to increase the production of endorphins [5]. Evidence suggests that endorphins upon binding to the cancer cells trigger apoptosis along with stimulating natural killer cells and helper T cells (TH). Complementary addition of LDN has shown encouraging results providing much-needed hope for fighting chronic crippling conditions [6].

## Case presentation

**Figure 1:**
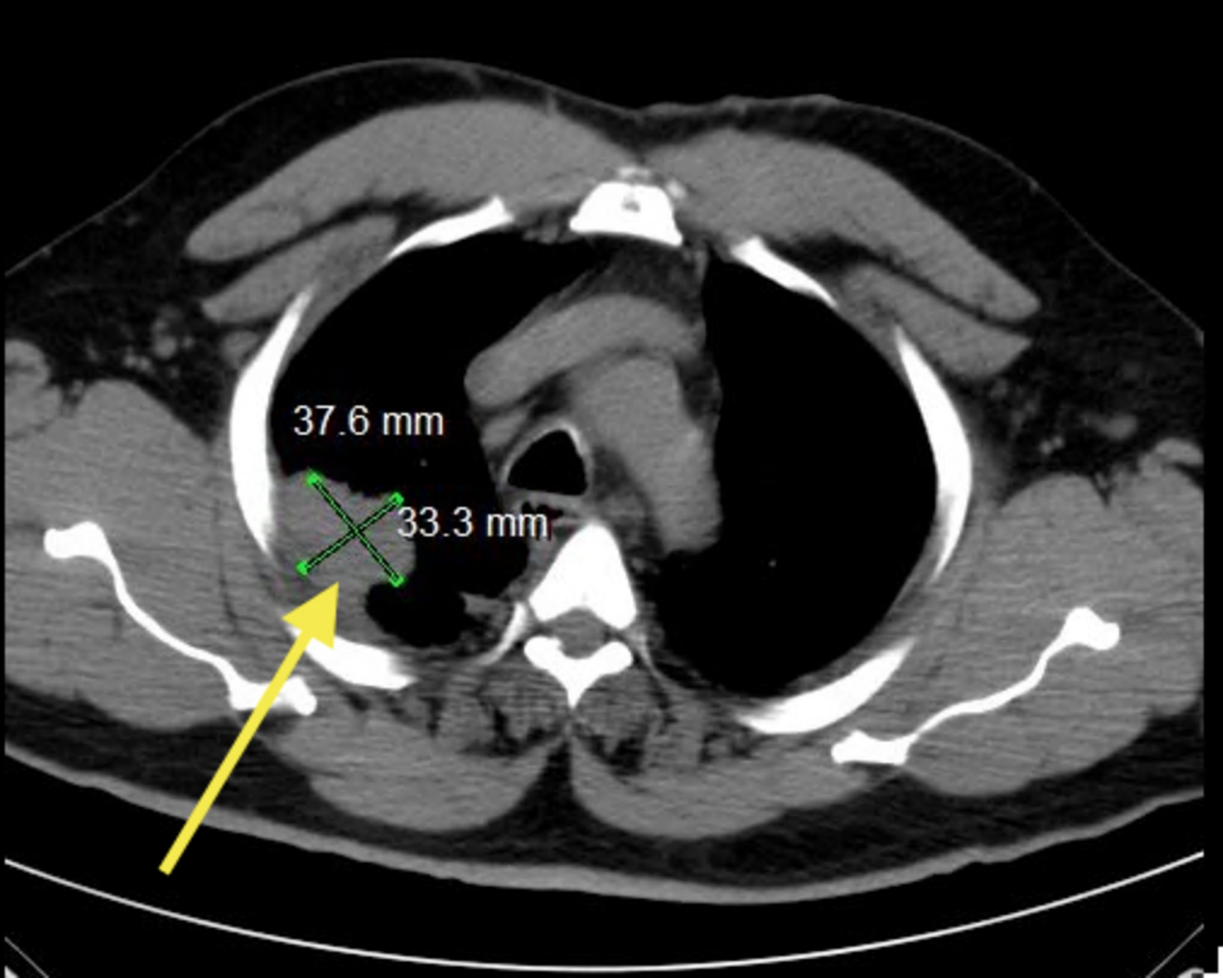
Computed tomography (CT) of the patient’s chest illustrating a pleural-based density in the right upper lobe measuring 3.3 cm x 3.7 cm (yellow arrow).

**Figure 2:**
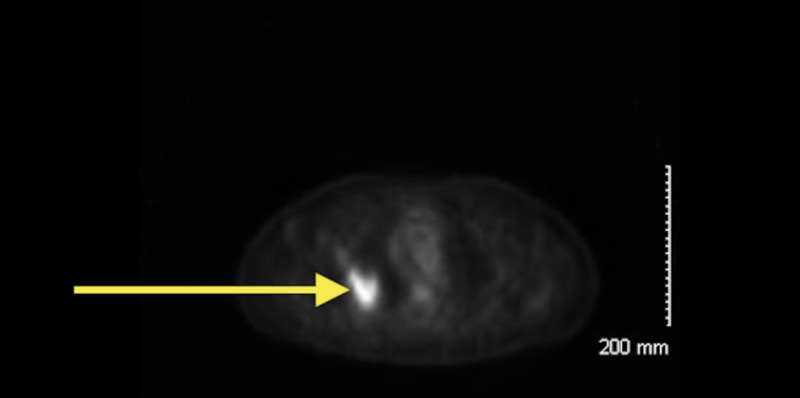
Positron emission tomography (PET) of the patient’s chest illustrating spiculated hypermetabolic mass in the right upper lobe of the lung consistent with primary lung tumor (yellow arrow).

A 50-year-old male who is an established patient of ours underwent preoperative evaluation in August 2013, which revealed an abnormality not present in the imaging taken in 2011. His chest X-ray revealed a prominence in right hilum and a density along the right pleura towards the right upper lobe. Later, the patient underwent noncontrast computed tomography (CT) of the chest depicting pleural-based mass measuring 3.3 cm x 3.7 cm in the right upper lobe (Figure 1). Biopsy performed a few weeks after imaging showed adenocarcinoma, poorly differentiated with a solid and single cell pattern. Molecular genetics was positive for thyroid transcription factor-1 (TTF-1), which is found in type II pneumocytes and Clara cells. Evidence suggests that TTF-1 expression is associated with better overall survival [7]. A full body positron emission tomography (PET) revealed a hypermetabolic spiculated 4.4 cm x 3.2 cm mass in the right upper lobe of the patient (Figure 2). Also, a pathologic hypermetabolic right suprahilar lymph node measuring 1.8 cm x 2.4 cm. was noted. The standardized uptake values (SUVs) were 12 and 5.8, respectively. On November 25, 2013, the patient underwent right upper lobe lobectomy and frozen section determined tumor, node and metastases (TNM) classification to be T3, N1, and Mx.  

After resection, the patient began concurrent chemo-radiotherapy with cisplatin and pemetrexed on January 15, 2014. He declined further chemotherapy treatments after the second treatment session citing intolerable side effects such as nausea, vomiting, fatigue, and malaise. However, he completed the radiation regimen, which lasted for a total of six weeks.   

In subsequent weeks and months, the patient faced numerous challenges including several admissions for pneumonia, fluid overload, and bronchospasms. Later, he was admitted for pulmonary embolism, which led to the addition of rivaroxaban for life and placement of an inferior vena cava (IVC) filter. The patient has a history of permanent tracheostomy for chronic hypercapnic respiratory failure in 2006 along with a radical prostatectomy due to a Gleason 7 prostate carcinoma in 2008.  Subsequently, after many positive antidotal cases in our practice utilizing LDN, a decision was made to try and start the patient on this complementary therapy. Therefore, he was started on LDN 4.5 mg HS on July 23, 2014. Imaging performed following LDN has been unremarkable. More specifically, a PET scan performed on October 27, 2017, and computed tomography (CT) of chest/abdomen/pelvis with contrast performed on April 25, 2018, have been negative for evidence of any recurrence. The patient is currently on rivaroxaban, amlodipine, atorvastatin, clonidine, insulin lispro, insulin glargine, losartan, metoprolol, prednisone, montelukast, diltiazem, and LDN. 

## Discussion

Naltrexone, an opioid antagonist, is primarily used in patients with alcohol or opioid dependence. Use of LDN is gaining popularity for managing patients battling various immunologically driven ailments. Naltrexone and its active metabolite, 6-β-naltrexol, are reversible competitive antagonists at μ-opioid (MOR) and κ-opioid receptors (KOR) [5, 8]. The standard dose of naltrexone, 50-150 mg, prevents inhibition of gamma-aminobutyric acid (GABA) receptor along with the prevention of dopamine release [9]. Evidence suggests that naltrexone, when used in low dose (1.75-4.5mg), shows inhibitory properties at the opioid receptors enabling the body to increase the production of endogenous opioids (endorphins) along with upregulating the immune system [9].  

Low dose naltrexone has been a part of various completed research and clinical trials along with the ongoing phase I, II, and III trials, which show promising results. Research suggests that LDN blocks opioids and blocks endogenous opioids such as endorphins only for a brief amount of time due to its low dose [5]. A transient deficit of endorphins stimulates the hypothalamus to create a rebound effect, which signals the body to increase the production of endorphins. Evidence suggests that this rebound effect triggers an increase in the production of opioid receptors, thereby increasing the sensitivity of these receptors, and also increasing the production of endorphins to compensate for the transient deficit of endorphins [9-10]. Consequently, increased levels of endogenous opioids play a vital role in promoting healing, inhibiting cell growth, and reducing inflammation among others [11].   

Research suggests that the LDN is metabolized by the liver and eliminated from the body within 3-4 hours. Despite inhibiting endorphins for a short amount of time, its effects can be felt up to 18-20 hours [5, 11]. During which, elevated endorphins play a central role in the downregulation of pro-inflammatory cytokines such as interleukin 6 (IL-6) and interleukin 12 (IL-12) along with tumor necrosis factor α (TNF-α) and nuclear factor κB (NF-κB) [12]. Furthermore, LDN’s inhibitory action at the opioid receptor also leads to an increase in the production of opioid growth factor (OGF) also known as metkephalin (MET) and opioid growth factor receptor (OGFr) via the positive feedback mechanism [8, 10]. Moreover, evidence suggests that OGF and OGFr play a significant role in regulating cell growth by altering transition from the gap 1 (G1) to the synthesis phase (S phase) of the cell cycle via the cyclin-dependent kinase inhibitor 2A (p16) and the cyclin-dependent kinase inhibitor 1 (p21) [9, 12]. Finally, LDN blocks toll-like receptor 4 (TLR-4) signaling to the glial cells thus decreasing their activation. In turn, inactive glial cells cannot activate pro-inflammatory cytokines resulting in decreased neuro-inflammation [5].    

Although there are many treatment options available, selecting an appropriate regimen is dependent on the stage and type of malignancy. Non-small cell lung cancer (NSCLC) can be treated with the standard of care regimen which includes surgery, radiation, chemotherapy, target therapy, and immunotherapy [13]. Those diagnosed with stage IIIA NSCLC are treated with surgery, if resectable, followed by chemotherapy, and radiation. Currently, in the USA, chemotherapy regimen of cisplatin and etoposide (CE) and weekly carboplatin and pacliltaxel are most commonly used in patients with stage IIIA NSCLC [14]. Pemetrexed, a newer agent, and cisplatin (PC) are options for nonsquamous cell carcinoma. PROCLAIM phase III trial, evaluated PC with radiation to CE with radiation, and the results were similar except PC resulting in lower incidence of neutropenia [15]. Evidence suggests that administration of chemotherapy and radiation together improves outcome compared to subsequent administration of a therapy [16].

Lung cancer is the most common cause of cancer mortality worldwide and recent estimates suggest the incidence of lung cancer to be 13.5% [17]. The percentage of all cancer deaths due to lung cancer is estimated to be 25.3%. The American Society of Clinical Oncology and the American Cancer Society predict a five-year survivor rate for stage IIIA to be 14%-36% [18-19].

## Conclusions

Physical, emotional, and psychologic impact of cancer on the patient and their families is challenging to quantify. So in light of statistics and our patient’s comorbidities of chronic respiratory failure, asthma, and tracheostomy, his response to LDN is worth documenting and further investigating. His inability to tolerate chemotherapy further strengthens the argument that LDN may have served a higher purpose than just being a simple alternative with minimal benefit. As immunotherapies for many disease states rapidly evolve, LDN at the very least deserves more attention by the medical and research communities. Some obstacles may include lack of interest by big pharmaceutical companies and lack of education regarding this off-label medicine once FDA approved for alcohol and heroin addiction at higher and less tolerated doses. Finally, LDN should not be taken concurrently with opioids because LDN can nullify the effects of narcotics.
